# Hydrophilic nanofibers with aligned topography modulate macrophage-mediated host responses via the NLRP3 inflammasome

**DOI:** 10.1186/s12951-023-02024-9

**Published:** 2023-08-14

**Authors:** Yiming Ren, Yi Chen, Wei Chen, Haotian Deng, Peiqi Li, Yubo Liu, Cangjian Gao, Guangzhao Tian, Chao Ning, Zhiguo Yuan, Xiang Sui, Shuyun Liu, Quanyi Guo

**Affiliations:** 1 School of Medicine, Nankai University, Tianjin, 300071 China; 2 Institute of Orthopedics, First Medical Center, Beijing Key Laboratory of Regenerative Medicine in Orthopedics, Key Laboratory of Musculoskeletal Trauma and War Injuries PLA, Chinese PLA General Hospital, No. 28 Fuxing Road, Haidian District, Beijing, 100853 China; 3 Plastic Surgery Hospital, Chinese Academy of Medical Sciences and Peking Union Medical College, 33 Badachu Road, Shijingshan District, Beijing, 100144 China

**Keywords:** Topography, Hydrophilicity, Macrophage, NLRP3 inflammasome, Immunomodulation, Peritendinous adhesion

## Abstract

Successful biomaterial implantation requires appropriate immune responses. Macrophages are key mediators involved in this process. Currently, exploitation of the intrinsic properties of biomaterials to modulate macrophages and immune responses is appealing. In this study, we prepared hydrophilic nanofibers with an aligned topography by incorporating polyethylene glycol and polycaprolactone using axial electrospinning. We investigated the effect of the nanofibers on macrophage behavior and the underlying mechanisms. With the increase of hydrophilicity of aligned nanofibers, the inflammatory gene expression of macrophages adhering to them was downregulated, and M2 polarization was induced. We further presented clear evidence that the inflammasome NOD-like receptor thermal protein domain associated protein 3 (NLRP3) was the cellular sensor by which macrophages sense the biomaterials, and it acted as a regulator of the macrophage-mediated response to foreign bodies and implant integration. In vivo, we showed that the fibers shaped the implant-related immune microenvironment and ameliorated peritendinous adhesions. In conclusion, our study demonstrated that hydrophilic aligned nanofibers exhibited better biocompatibility and immunological properties.

**Supplementary Information:**

The online version contains supplementary material available at 10.1186/s12951-023-02024-9.

## Introduction

Biomaterials are widely used for tissue regeneration, repair and replacement, as well as to achieve specific functions, such as the promotion of hemostasis and the prevention of adhesion [1, 2]. Appropriate host responses and biomaterial compatibility are prerequisites for the successful performance of these materials [3, 4]. Additionally, interactions between cells and biomaterials are also important. The physical, chemical, biological and mechanical cues of these materials have considerable effects on cell behaviors [5–7]. In this context, manipulating the behavior and fate of cells by altering the material properties is an attractive and promising approach for achieving or improving the functions of such materials. Controlling the immune response to implants is of great importance, and the ecological niche of the immune microenvironment is critical.

The prevention of unfavorable foreign body reactions (FBRs) is one of the main challenges in the field of bioengineering. Inflammation interferes with the integration of implants and communication between tissues and biomaterials. Therefore, immunomodulation is a new approach for mitigating foreign body reactions that has been used to not only improve the outcome after implantation but also modulate the immune microenvironment for therapeutic purposes [8]. Macrophages are a part of the innate immune system that act as key mediators between the host and implanted materials. They participate in cascade reactions in response to tissue injury and FBRs in response to implants [4]. Moreover, the roles of macrophages in the regeneration and repair of tissues are increasingly being recognized [9, 10]. Macrophages are highly plastic. They can polarize into different phenotypes under different environmental conditions and in response to different stimuli [11]. Macrophages have classically been divided into two types: the proinflammatory M1 type and the anti-inflammatory M2 type. M2-type macrophages are also thought to facilitate tissue regeneration and repair [12]. To date, some studies have explored the interactions between materials and macrophages, and consequently, material design strategies that promote immunomodulation have been proposed [13]. However, the detailed understanding of biomaterial-immune system interactions is still limited.

Electrospinning fibers have a unique spatial structure that mimics the extracellular matrix, such as tendon tissues [14]. Their pores allow the transport of nutrients and wastes. Therefore, electrospinning technology is widely used in the fields of tissue repair and drug delivery. However, FBRs to fibers inhibit their performance [15], and these reactions are thought to be initiated due to the composition and structure of the fibers [16, 17]. Currently, the mechanisms underlying the crosstalk between fibers and macrophages are still unclear and have attracted our attention. Here, we employed the widely used polyethylene glycol (PEG) and polycaprolactone (PCL) as paradigms to attempt to elucidate the effects of fibrous material composition and structure on immune responses. This study may guide the future design of biomaterials.

**Fig. 1:**
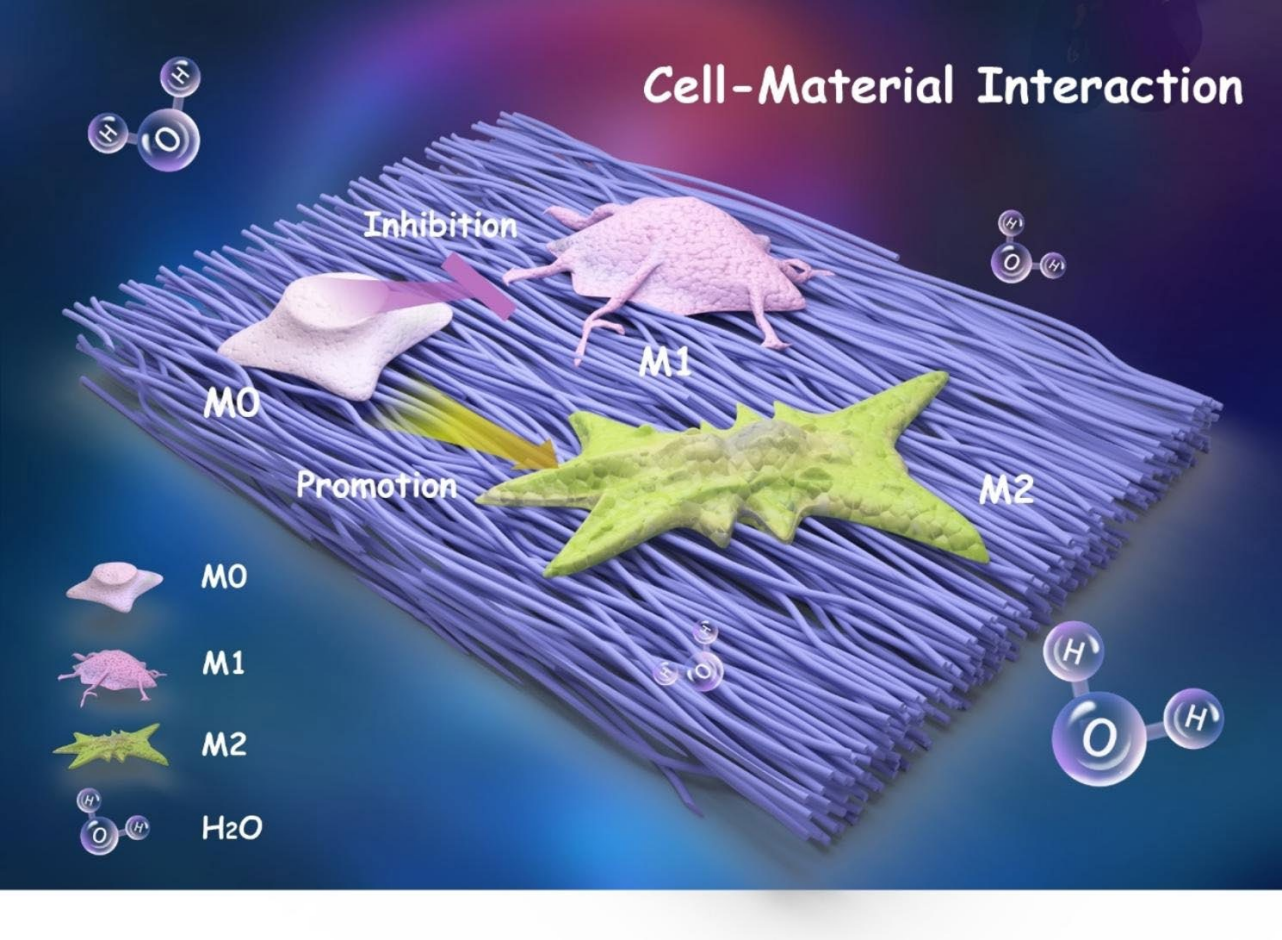
Schematic diagram showing a shift in macrophage polarization when they are cultured on aligned nanofibers

Hydrophilic nanofibers with an aligned topography were developed based on the axial electrospinning technique. We explored their effects on macrophage behavior and tried to elucidate the underlying mechanisms (Fig. 1). We originally identified that NOD-like receptor thermal protein domain associated protein 3 (NLRP3) inflammasome is a participant in nanofiber-mediated immune responses. Intracellular danger sensors activate caspase-1 to facilitate the maturation and release of inflammatory factors [18]. This response is also intrinsically associated with tissue repair, mediating the initiation of inflammation and regeneration, which in turn leads to chronic inflammation and fibrosis [19, 20]. Therefore, the inflammasome is a bridge-like regulator of inflammation and tissue repair. Our studies suggested that regulating inflammasome activation is an effective strategy that would allow fibrous materials to achieve implant integration and immune modulation, which was demonstrated in an animal model of postinjury peritendinous adhesions.

## Materials and methods

### Preparation of the nanofibers

Aligned nanofibers were prepared by a high-speed axial electrospinning method. Hexafluoroisopropanol (Aladdin) was used as the spinning solvent. PCL (80 kDa) and PEG (6 kDa) were purchased from Sigma‒Aldrich. A 9% PCL polymer solution was used to prepare PCL nanofibers. A solution of 3% PEG and 9% PCL was mixed to prepare L-PECL nanofibers. A 9% PEG and 3% PCL solution were mixed to prepare H-PECL nanofibers. The prepared solutions were added to a 10 ml syringe that was connected to an 18G electrospinning nozzle. The syringe was mounted on the electrospinning device (Yongkang Leye Company, Beijing), and the operation was performed in a clean environment at room temperature. In this study, 13 kV was applied to the spinning solution, and − 8 kV was applied to the tinfoil-covered axial receiver with a high rotation speed of 4500 rpm. The pump speed and receiving distance were adapted as needed. The disordered nanofiber membranes were collected by adjusting the rotation speed to 20 rpm. After spinning was completed, the membranes were air-dried on an ultraclean table.

### Characterization of the nanofiber membranes

The nanofiber films were fixed to the test bench with conductive double-sided tape. After spraying with gold, the samples were visualized by field emission scanning electron microscopy (FESEM; FEI-NOVA NanoSEM 230, USA). Image J was then used to determine the fiber diameters and orientations according to the SEM images. Fourier transform infrared (FTIR) spectroscopy (Nicolet IS5, Thermo Fisher, USA) was used to characterize the nanofiber films in the range of 525–4000 cm − 1. To determine the hydrophilicities of the different nanofibers, the water contact angles of the membranes were measured using a contact angle goniometer (Dataphysics-OCA20, Germany). Three replicates were performed for each group.

### Cell isolation and culture

Bone marrow-derived macrophages (BMDMs) were harvested from six-week-old C57BL/6 N mice according to our previously described protocol [21]. The animal experiment was approved by the Institutional Animal Care and Use Committee of the PLA General Hospital (SCXK No. 2019-0018) and followed the US National Research Council’s Guide for the Care and Use of Laboratory Animals. Briefly, after the mice were sacrificed, the bone marrow was removed from the femur and tibia by washing under sterile conditions. After filtration through a sterile 100 μm filter, the cells were treated with erythrocyte lysis buffer. The cells were then centrifuged and cultured in DMEM (Gibco) supplemented with 10% inactivated fetal bovine serum (FBS; Gibco), 100 U/ml penicillin‒streptomycin (Gibco) and 35 ng/ml M-CSF (Peprotech). After 7 days, the BMDMs were harvested for subsequent experiments. The PCL, L-PECL and H-PECL nanofibrous membranes were cut into circular pieces approximately 1.5 cm in diameter. After irradiation with UV light, they were washed three times with PBS for at least 5 min each time. The nanofiber pieces were placed in 24-well plates. Three replicates were performed for each group. BMDMs were seeded at a density of 1 × 10^5^ cells/well and incubated at 37 °C in 5% CO_2_ for subsequent experiments.

### Cytoskeleton staining

To visualize cell spreading on the nanofibers, BMDMs were cultured on PCL, L-PECL, H-PECL nanofibers and TCP for 48 h. For all experiments, cells cultured on TCPs were used as controls. Three replicates were performed for each group. After washing twice with PBS and fixing with 4% paraformaldehyde, the cells were permeabilized with 0.5% Triton X-100 for 20 min. FITC-labeled phalloidin solution (Beyotime, China) was added to stain the cytoskeleton. After incubation for 30 min in the dark, the cells were stained with 4,6-diamidino-2-phenylindole (DAPI; Sigma‒Aldrich) solution for 5 min. After washing and sealing, the cell morphology was observed and photographed with a confocal microscope (Leica, Japan). In order to analyze the formation of stress fibers, a five-point scale was used to measure the degree of actin stress fiber [22].

### In vitro immunofluorescence staining

For cellular immunofluorescence staining, nanofiber membranes from each group that had been seeded with cells were placed in tissue culture plates, washed twice with PBS and then fixed with 4% paraformaldehyde for 15–20 min. Then, 0.5% Triton X-100 was added for 30 min of incubation. Bovine serum albumin (BSA) (3%) was added to the cells, which were incubated for 30 min for blocking. Subsequently, a primary antibody was added for incubation overnight at 4 °C. Then, the nanofiber membranes were washed three times with PBS and incubated with a fluorescently coupled secondary antibody for 1 h. DAPI staining solution was added dropwise, and incubation was continued for 5 min. Fluorescence confocal microscopy was used to observe and photograph the samples after they were washed with PBS. Three replicates were performed for each group, and three images of the field of view were acquired for each sample. Image J was used to obtain the average relative fluorescence intensity, by adjusting the threshold and confirming a suitable algorithm. Primary antibodies against iNOS (Abcam), Arg-1 (Abcam), and ASC (Novus) were used. Dilution of the antibodies are listed in Supplementary Table S6. The antibodies were diluted with the antibody dilution containing 3%BSA.

### BMDM inflammasome activation

BMDMs (1 × 10^5^) were seeded on PCL, L-PECL and H-PECL nanofiber membranes (diameter of 14 mm) and cultured in an incubator at 37 °C in 5% CO_2_. Three replicates were performed for each group. After 48 h of incubation, 100 ng/ml LPS (Sigma) was added to stimulate the BMDMs for 4 h, and later, the BMDMs were incubated with 10 mM nigericin (MCE) for 30 min. The culture supernatants were collected to determine IL-1β secretion. Total protein was extracted from the BMDMs and subjected to Western blotting to analyze the expression of the NLRP3 inflammasome and pathway-related proteins. Another portion of cells was fixed with 4% paraformaldehyde (PFA) for 10 min, and then immunofluorescence staining for ASC (Novus) was performed. The oligomerization of ASC, which can indicate inflammasome assembly, was observed by fluorescence microscopy. ASC speck quantification was performed using open source QuPath software [23].

### ELISA

An ELISA kit (Invitrogen) was used to measure the IL-1β levels in the macrophage sample supernatants according to the manufacturer’s instructions.

### Subcutaneous implantation

To assess the effect of the nanofiber membranes on macrophage recruitment and polarization, we established an animal subcutaneous implantation model in 6-week-old SD rats. All the protocols were approved by the Institutional Animal Care and Use Committee of the PLA General Hospital (SCXK No. 2019-0018) and followed the US National Research Council’s Guide for the Care and Use of Laboratory Animals. The rats were randomly divided into three groups, namely, the PCL group, L-PECL group and H-PECL group, with four rats in each group. After the rats were subjected to general anesthesia with 1% pentobarbital, the dorsal hair was shaved, and the skin was disinfected. An approximately 1 cm cut was made along the centerline of the back, and a nanofiber membrane (diameter of 1 cm) was placed in the subcutaneous space according to the grouping. All nanofiber membranes were sterilized by UV irradiation before implantation. Three different types of membranes were placed in each mouse. Finally, the dorsal incision was sutured. Twenty-four hours later, the nanofiber membranes were extracted and washed with PBS. After fixation in 4% paraformaldehyde, immunofluorescence staining was performed as described above. Three images of the field of view were acquired for each sample. The primary antibodies included anti-CD86 (Novus), anti-CD206 (Cell Signaling), anti-CD68 (Novus) and anti-ASC (Novus). Dilution of the antibodies are listed in Supplementary Table S6. The antibodies were diluted with the antibody dilution containing 3%BSA.

### Achilles tendon surgery in animals

We used SD rats to establish a model of Achilles tendon injury to study postoperative adhesions. The animal protocols were approved by the Institutional Animal Care and Use Committee of the PLA General Hospital (SCXK No. 2019-0018) and followed the US National Research Council’s Guide for the Care and Use of Laboratory Animals. Adult male SD rats weighing 200 ± 50 g were randomly assigned to four groups: the control group, PCL group, L-PECL group, and H-PECL group. Four replicates were performed for each group. After anesthesia, shaving and disinfection, an approximately 1 cm longitudinal incision was made on the inner side of the right Achilles tendon to expose the tendon. The tendon was partially transected at a distance of 0.5 cm from the Achilles tendon stop and then sealed according to a modified Kessler method with 6 − 0 nylon (Ethicon). The three types of nanofibrous membranes were wrapped around the sutured tendons after being sterilized. Then, the skin was sutured. Uncoated Achilles tendons were used as controls. Three weeks later, the rats were sacrificed, and the adhesions were assessed by macroscopic observations and tissue staining. Scoring rules for macroscopic adhesions and shapes assessments are listed in Supplementary Tables S2 and S3.

### Histological staining and evaluation

The collected tendons were immediately embedded in frozen sectioning medium (Leica) and then cut into 5-mm frozen sections using a sectioning machine (Leica CM1950). Hematoxylin-eosin (H&E) and Masson staining were subsequently performed to observe adhesions and collagen fibrosis formation around the Achilles tendon under a light microscope (Leica Microsystems, Wetzlar, Germany). Histological adhesions and inflammatory responses were quantified according to a 4-point scale as shown in Supplemental Tables S4 and S5.

Additional experimental procedures are provided in the Supplementary Information.

### Statistical analysis

The data are presented as the mean ± standard deviation, and the data were analyzed by two-tailed Student’s t test or one-way ANOVA with Tukey’s post hoc tests. Statistical analysis was performed using GraphPad Prism 8.0 software. *p < 0.05 represents the threshold for statistical significance.

## Results

### Successful fabrication of highly aligned nanofibers

**Fig. 2:**
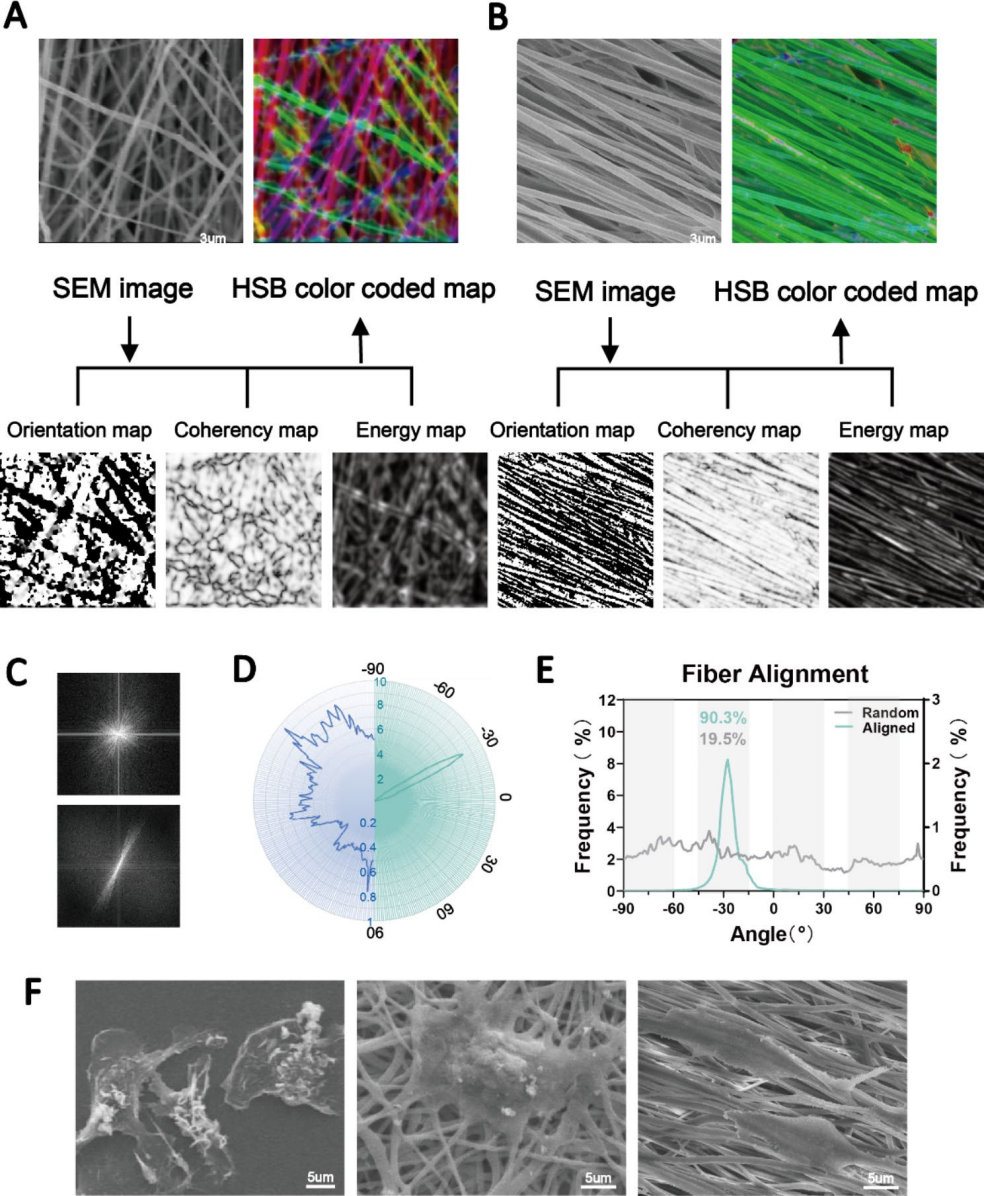
Fabrication of aligned and disordered electrospun nanofibers. (**A, B**) SEM images and the orientation, energy, coherency and HSB color-coded maps of aligned and random PCL nanofibers. (**C, D, E**) Determination of the orientations of aligned and random fibers as visualized by Fourier and radar charts. (**F**) SEM images showing macrophages different morphologies attached to TCP substrates (left panel), random nanofibers (middle panel) and aligned nanofibers (right panel)

High-speed rotation of the electrospun axial receiver allows for the generation of aligned fibers. Here, we prepared highly aligned PCL fibers in contrast to random fibers. The SEM images showed that both the aligned and random nanofibers had smooth surfaces and were uniform in diameter. We evaluated the orientation of the fibers based on the structure tensors using Orientation J [24]. Local orientation was analyzed and is shown in Fig. 2A. To effectively include only the angular values of the fiber edges, we utilized the isotropic properties (coherency and energy) to distinguish between the uniform and edge areas. The color-coded maps are composites of the orientation, coherency and brightness maps, and they indicate the oriented structures of the fibers. Furthermore, we quantified the orientation of the aligned and random fibers (Fig. 2B-D), which exhibited significantly different patterns of angle distribution. A total of 90.3% of the aligned fibers were oriented from − 45° to − 15°, while the random fibers were oriented in a nonfocused manner (Fig. 2B). The Fourier analysis and radar charts showed similar results, where the orientation of the aligned fibers was in focus (Fig. 2C, D, E).

Subsequently, we plated bone marrow-derived macrophages on the two fibers and on TCP as a control. The results showed that the fiber orientation significantly affected the extension of the cells. We observed macrophages with an elongated morphology on the aligned fibers and polygonal macrophages on the random fibers (Fig. 2F). These results indicated the successful preparation of highly aligned nanofibers. Angelina et al. reported that aligned PCL fibers more strongly induced M2 macrophage polarization than random fibers [17]. However, inflammatory reactions to foreign bodies were still inevitable. Therefore, this study focused on further improving the physicochemical properties of the fibers to achieve better immune tolerance and regulation. To unify the variables, only aligned fibers were used for in vitro and in vivo assessment.

### Characterization of hydrophilic nanofibers with an aligned topography

**Fig. 3:**
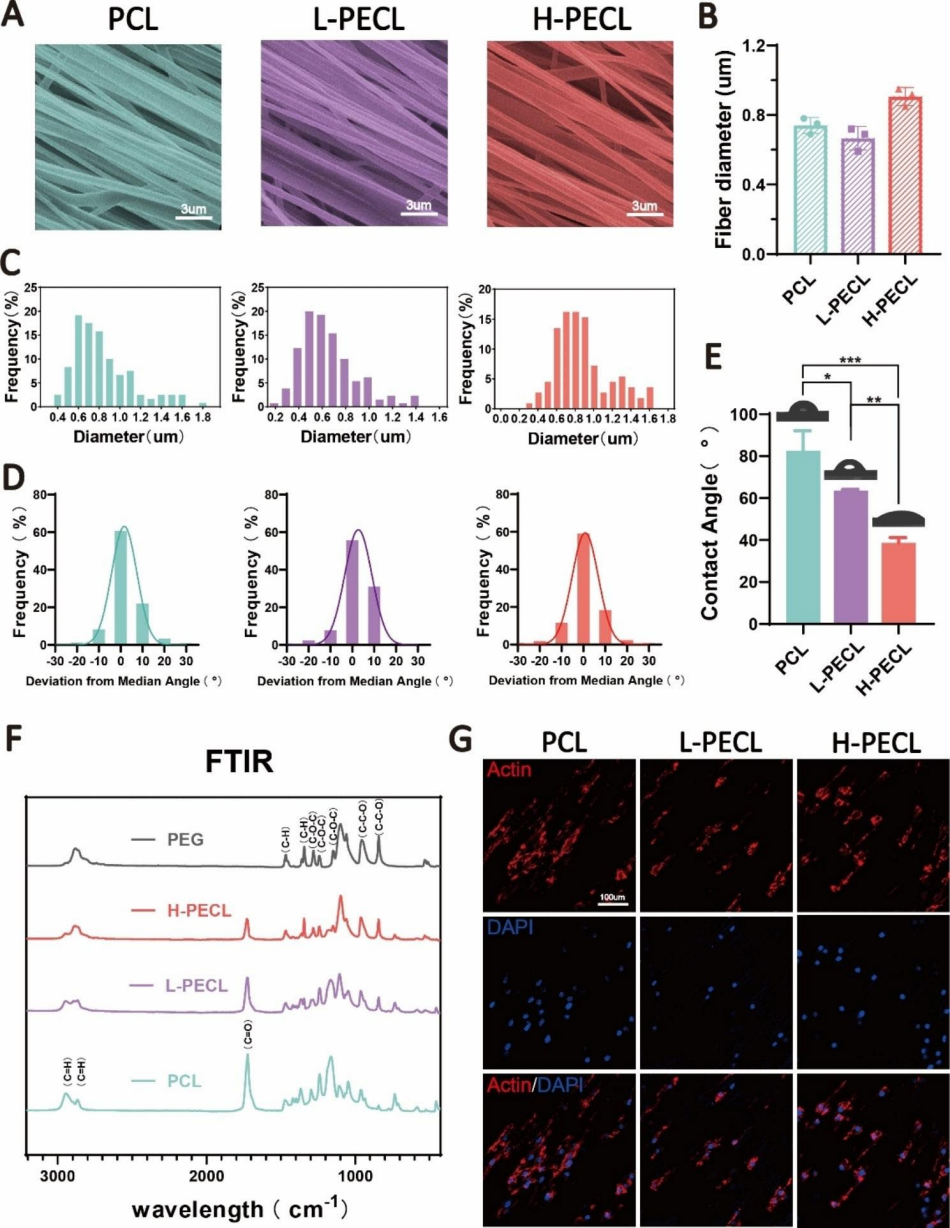
Characterization of PEG-modified aligned fibers with different degrees of hydrophilicity. (**A**) SEM images, (**B**) diameter distribution and (**C**) average diameters of the three types of nanofibrous membranes (from left to right PCL, L-PECL, and H-PECL). (**D**) Determination of the orientations and (**E**) water contact angles of the different types of membranes (n = 3). (**F**) Fourier transform infrared (FTIR) spectra of the PCL, L-PECL, and H-PECL nanofibers. (**G**) Cytoskeleton staining showing the oriented extension of macrophages along the nanofibers (Actin: red and nucleus: blue). The data are presented as the mean ± standard deviation of three experimental replicates. Statistical analysis was performed by one-way ANOVA with Tukey’s post hoc analysis. *p < 0.05; **p < 0.01, ***p < 0.001; ns indicates no significance

To modify the surface properties of the nanofibers, we prepared aligned nanofibers by incorporating PCL and PEG by axial electrospinning. H-PECL and L-PECL refer to the nanofibers with high and low PEG proportions, respectively, which had different degrees of hydrophilicity [25, 26]. The SEM images showed that the nanofibers in each group were relatively uniform in size, with smooth surfaces and no bead-like structures. Additionally, the nanofiber films were highly aligned (Fig. 3A). The diameters of the PCL, L-PECL, and H-PECL fibers were 0.74 ± 0.05 μm, 0.66 ± 0.07 μm, and 0.91 ± 0.05 μm, respectively (Fig. 3B, C). The H-PECL fibers had a larger diameter. We analyzed the orientation of the three types of nanofibers. The deviation angles were mostly concentrated in the range of -15° to 15° (Fig. 3D). This indicated that all of the membrane types were well oriented. Then, we measured their water contact angles (Fig. 3E), and the results suggested that the incorporation of PEG increased the hydrophilicity of the fibers. Moreover, the hydrophilicity increased with increasing PEG proportion. The chemical composition of the nanofibers was characterized by FTIR. With increasing proportions of PEG, the intensity of the C = O stretching band of PCL decreased, while the intensity of the C-O-C stretching band of PEG increased. The L-PECL and H-PECL fibers exhibited characteristics of both PCL and PEG (Fig. 3F).

To investigate the effect of nanofibers on macrophages, we cultured BMDMs on the three types of fibrous nanomembranes. We observed that the BMDM adhered to the fibers, and the macrophages extended along the direction of the fibers (Fig. 3G). This suggested that the nanofibers significantly affected the morphology of the macrophages. We then assessed the adhesion of macrophages to different nanofiber membranes by analyzing the formation of stress fibers. In general, the semiquantitative analysis of the stress fiber formation showed a decrease in stress fibers as the amount of PEG increased, as shown in Figure S1. This gap may be attributed to the non-adherent nature of PEG to proteins or cells.

### Nanofiber hydrophilicity alters the phenotype of macrophages

**Fig. 4:**
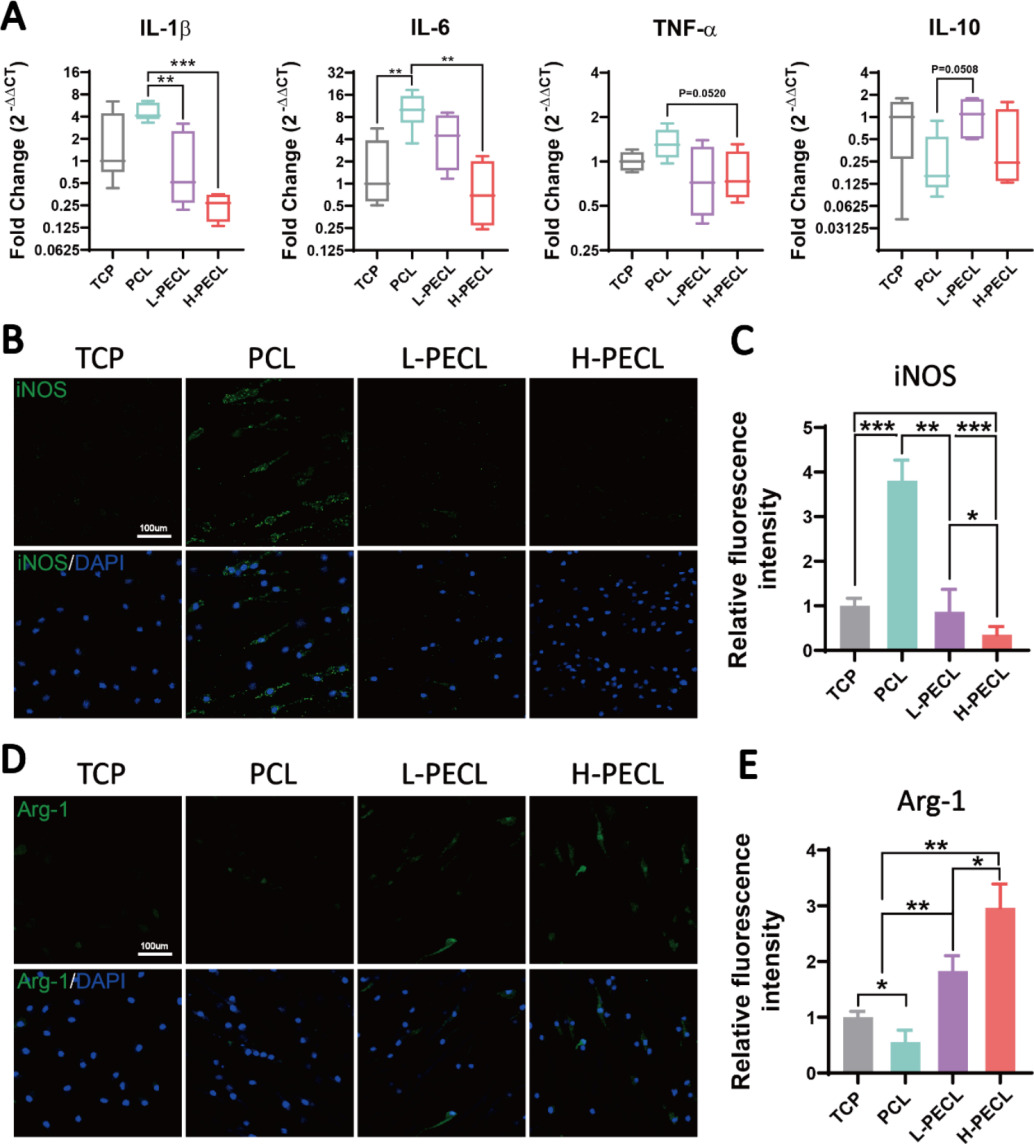
Changes in the polarization of macrophages cultured on different types of nanofibers. (**A**) RT–PCR was used to determine the gene expression of the proinflammatory factors IL-1b, IL-6, and TNF-α and the anti-inflammatory factor IL-10 in BMDMs cultured on different types of nanofibers. (**B, C**) Immunofluorescence analysis of iNOS, indicating M1 polarization (iNOS: green and nucleus: blue). (**D, E**) Immunofluorescence analysis of Arg-1, indicating M2 polarization (Arg-1: red and nucleus: blue). The data are presented as the mean ± standard deviation of three experimental replicates. Statistical analysis was performed by one-way ANOVA with Tukey’s post hoc analysis. *p < 0.05; **p < 0.01, ***p < 0.001; ns indicates no significance

We seeded BMDMs on each type of fibrous membrane to analyze the changes in macrophage phenotype. First, we measured the expression levels of inflammatory genes. Compared to the macrophages on the TCP control, the macrophages on PCL nanofibers showed significantly higher expression of the proinflammatory genes IL-1β and IL-6 and decreased expression of the anti-inflammatory gene IL-10 (Fig. 4A). This confirmed that PCL induced an inflammatory response in macrophages. As the proportion of PEG in the nanofibers increased, the expression of inflammatory genes gradually decreased. In the H-PECL group, the expression of IL-1β was even lower than that in the TCP group. This may be due to the hydrophilic and axial properties of the nanofibers.

Next, we analyzed the polarization of macrophages by immunofluorescence staining for the M1 phenotypic marker iNOS and the M2 phenotypic marker Arg-1. The results showed remarkable positive staining for iNOS in the PCL group. In contrast, the expression of iNOS was lower in the L-PECL and H-PECL groups (Fig. 4B, C). Arg-1 expression showed the opposite trend, and the highest expression was observed in the H-PECL group (Fig. 4D, E). These results suggested that PCL fibers induced macrophage polarization toward the proinflammatory M1 phenotype, whereas the incorporation of PEG reduced this shift and promoted macrophage polarization toward the anti-inflammatory M2 phenotype.

### *The NLRP3 inflammasome is the cellular sensor that is responsible for hydrophilic nanofiber-driven macrophage polarization*

**Fig. 5:**
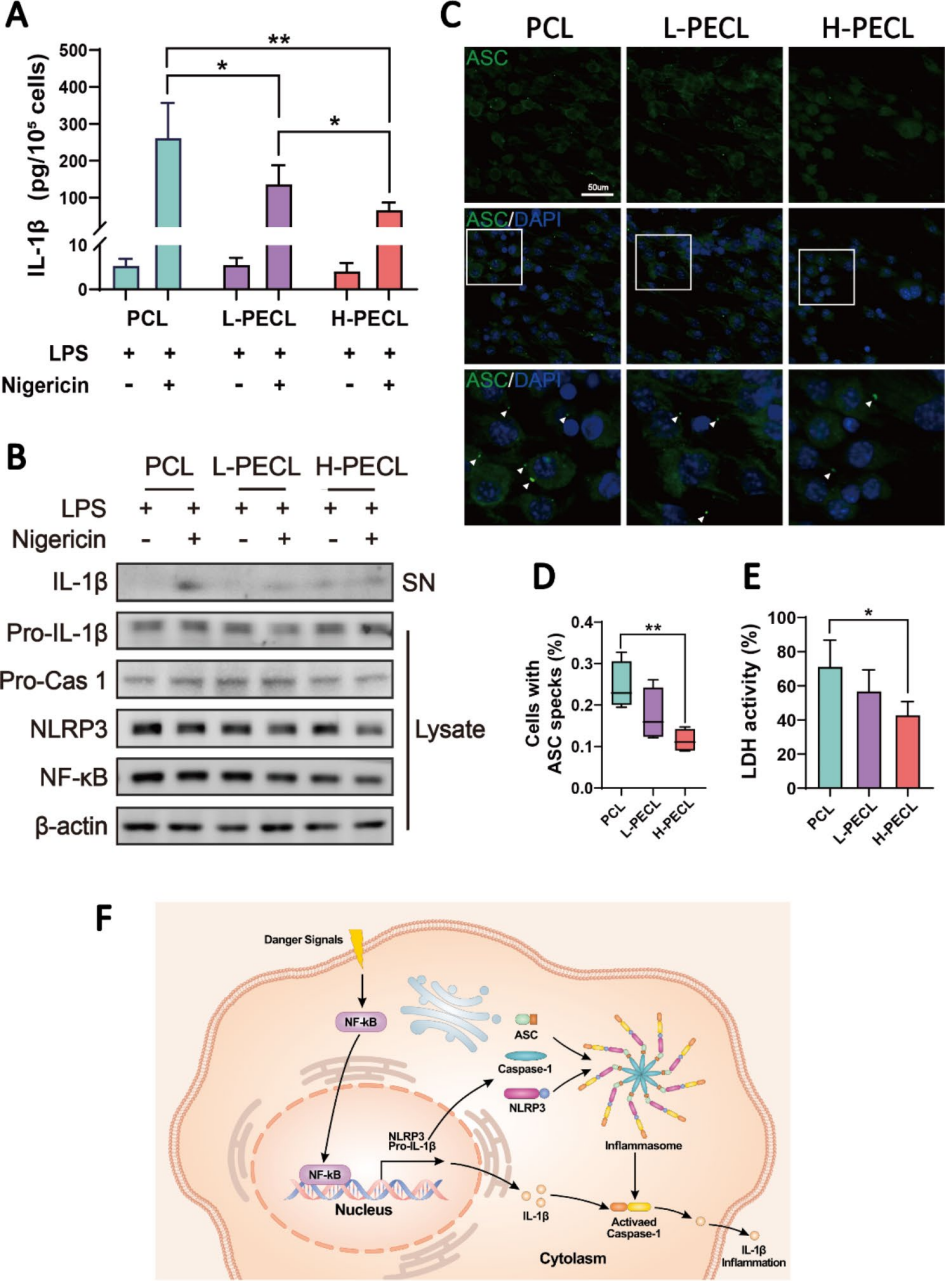
IL-1b release and NLRP3 inflammasome activation in macrophages cultured on different types of nanofibers. (**A**) ELISA was used to determine IL-1b release by BMDMs stimulated with LPS and nigericin. (**B**) Western blotting analysis of the expression of NLRP3 and related signaling proteins in BMDMs stimulated with LPS and nigericin. (**C**) Immunofluorescence analysis of ASC oligomerization in BMDMs (ASC: green and nucleus: blue), indicating inflammasome assembly. (**D**) Proportions of BMDMs with ASC specks. (**E**) Conceptual graphs of NLRP3 inflammasome activation and function. The data are presented as the mean ± standard deviation of three experimental replicates. Statistical analysis was performed by two-tailed Student’s t test and one-way ANOVA with Tukey’s post hoc analysis. *p < 0.05; **p < 0.01, ***p < 0.001; ns indicates no significance

According to the results described above, we can infer that the polarization of macrophages differentiated when they were cultured on different types of nanofibers. Accordingly, we proceeded to explore which pathways affected their polarization. From Fig. 4A, we observed a substantial difference in the expression of the proinflammatory gene IL-1β. Therefore, we measured the secretion of IL-1β by BMDMs cultured on each type of membrane by ELISA (Fig. 5A). However, we found that IL-1β was expressed at low levels in all the groups. Considering that the secretion of IL-1β is closely associated with inflammasome activation, we stimulated BMDMs with LPS and nigericin and subsequently measured IL-1β secretion. The ELISA results showed that more IL-1β was released by the macrophages in all groups after stimulation (Fig. 5A). Encouragingly, macrophages in the H-PECL group secreted less IL-1β than those in the other two groups, and the difference was statistically significant.

To further investigate the cellular sensor that is responsible for nanofiber-driven macrophage polarization, we analyzed the expression of the NLRP3 inflammasome and related proteins in the cytoplasm and supernatants of BMDMs. The results showed decreased expression of NLRP3 and NF-kb in the macrophages cultured on nanofibers with PEG, and the levels of IL-1b that were secreted into the supernatants were also reduced (Fig. 5B). Activation of the NLRP3 inflammasome involves the assembly of NLRP3, ASC and caspase-1 (Fig. 5F). Therefore, we performed additional immunofluorescence staining for ASC to visualize inflammasome assembly. Oligomerization of ASC was observed in response to LPS and nigericin treatment, as indicated by the white arrows in Fig. 5C. As expected, the number of ASC specks in the macrophages in the H-PECL group was the lowest (Fig. 5D). Inflammasome assembly in macrophages leads to pyroptosis. Consequently, lactate dehydrogenase (LDH) release by macrophages indicates the degree of pyroptosis [27]. The macrophages cultured on PEG-modified nanofibers exhibited lower levels of LDH activity, indicating fewer pyroptotic cells (Fig. 5E). These results indicated that PEG modified the surface properties of the nanofibers and inhibited the activation of the NLRP3 inflammasome in macrophages.

### *Subcutaneous macrophage-mediated immune responses and inflammasome activation*

**Fig. 6:**
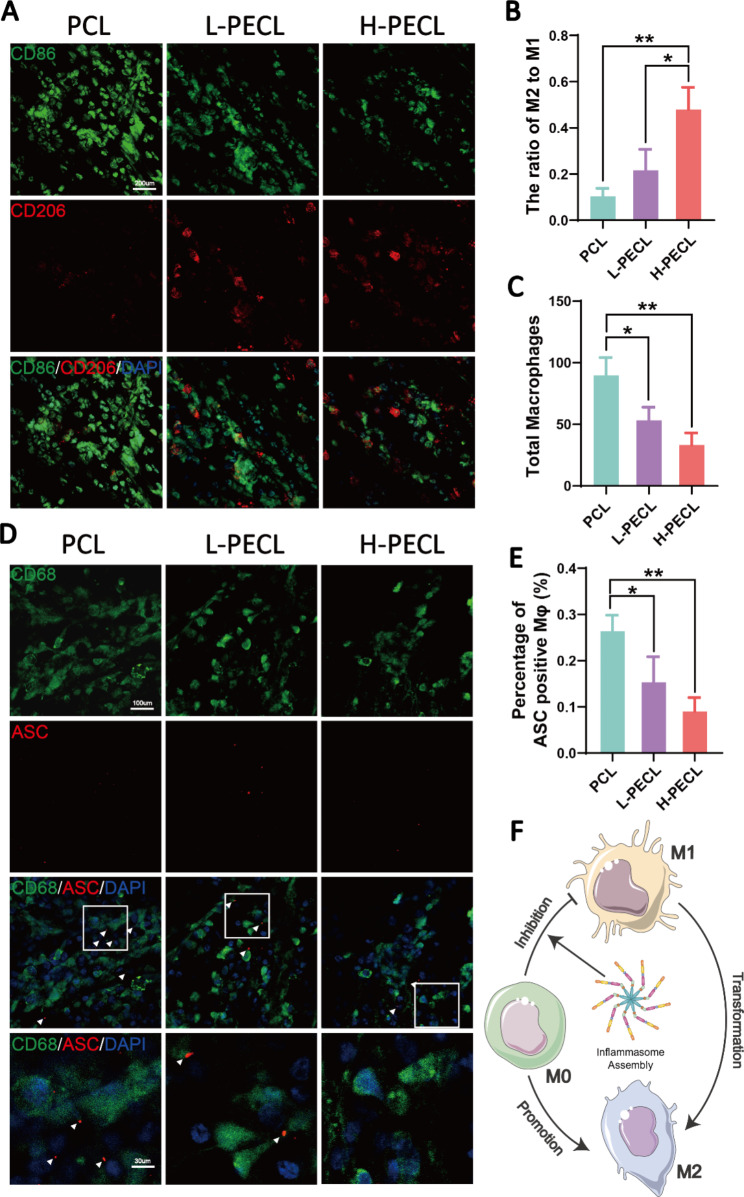
Subcutaneous macrophage recruitment, polarization and inflammasome activation. (**A**) Immunofluorescence analysis of CD86 and CD206, indicating macrophage recruitment and polarization, on different types of subcutaneous membranes (CD86: green, CD206: red, and nucleus: blue). (**B**) Statistical analysis of the ratios of M2 to M1 macrophages. (**C**) Statistical analysis of the total number of recruited macrophages. (**D**) Immunofluorescence images of subcutaneous ASC oligomerization in recruited macrophages (ASC: red, CD68: green, and nucleus: blue). (**E**) Proportions of macrophages with ASC specks. (**F**) Conceptual graphs of macrophage polarization. The data are presented as the mean ± standard deviation of three experimental replicates. Statistical analysis was performed by one-way ANOVA with Tukey’s post hoc analysis. *p < 0.05; **p < 0.01, ***p < 0.001; ns indicates no significance

We subcutaneously implanted the three types of nanofibrous membranes into model animals to explore the macrophage-mediated foreign body response. Immunofluorescence staining was performed to identify the macrophage phenotype. CD86 is a marker of M1 macrophages, while CD206 is a marker of M2 macrophages. As shown in Fig. 6, macrophage infiltration was observed in all three groups, with the highest infiltration in the PCL group (Fig. 6A, C). In contrast, the H-PECL group had the lowest extent of macrophage infiltration and the highest proportion of M2-type macrophages (Fig. 6B, C). These results suggested that the PCL fibers elicited a stronger early host immune response. In addition, most of the macrophages that were recruited to the nanofibrous membranes in the early stage exhibited a proinflammatory phenotype, while the incorporation of PEG into the nanofibers promoted macrophage polarization toward the anti-inflammatory phenotype. This helped to attenuate the foreign body response and enable proactive immune regulation.

Furthermore, we performed immunofluorescence staining for ASC to evaluate inflammasome activation in macrophages cultured on subcutaneously implanted nanofibers (Fig. 6D). CD68 is a pan macrophage marker. The results showed that subcutaneously implanted nanofibers recruited macrophages and initiated the assembly of inflammasomes. The lowest percentage of ASC specks was observed in the H-PECL group (Fig. 6E), suggesting that PEG-modified nanofibers could reduce inflammation by inhibiting inflammasome assembly during the early stages of implantation (Fig. 6F).

### *In vivo study: PEG-incorporated aligned nanofibrous membranes mitigate peritendinous adhesions*

**Fig. 7:**
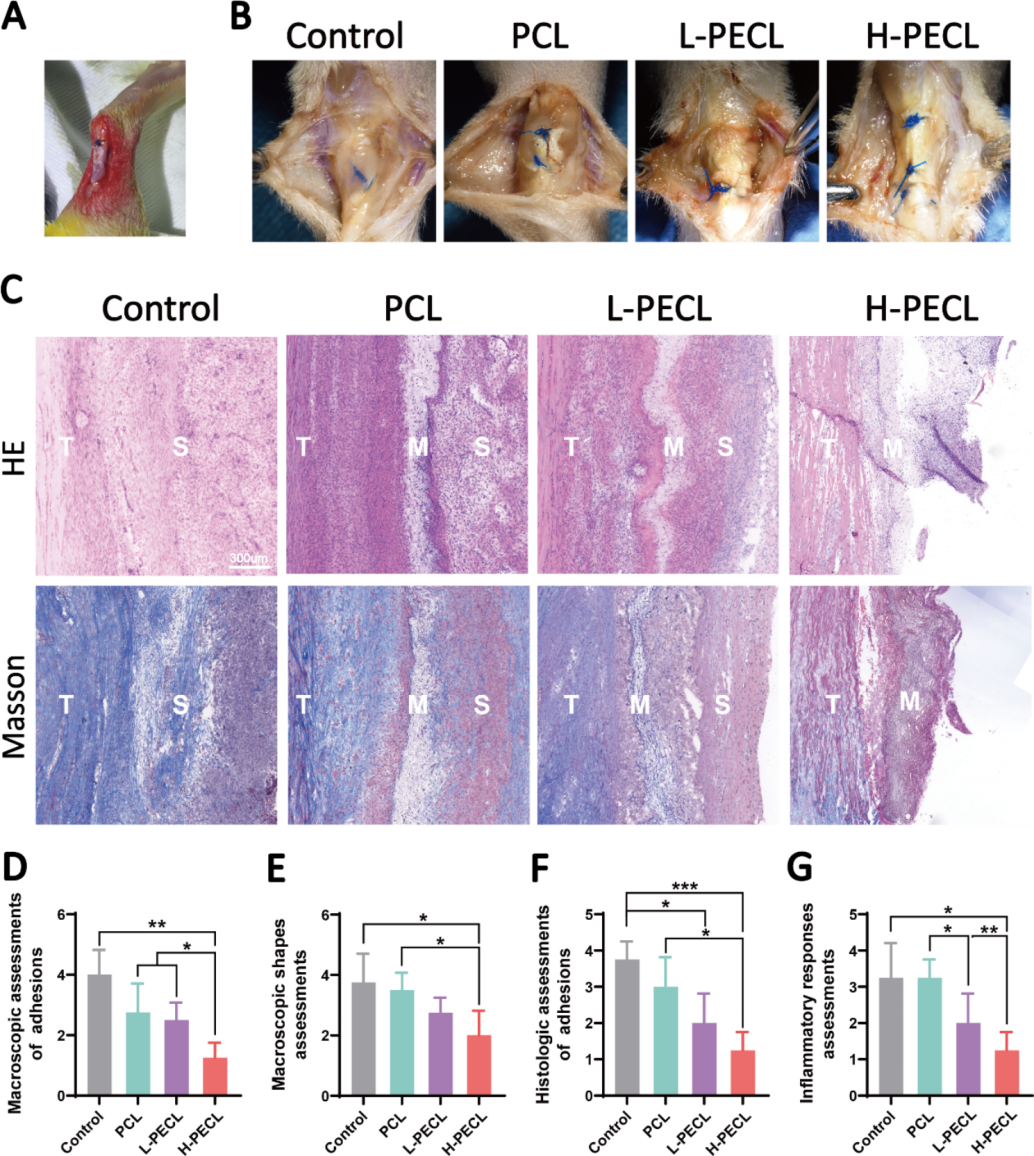
Macroscopic observations and histological staining of Achilles tendons treated with different types of nanofibers at 3 weeks post-surgery. (**A**) Surgical procedure by which the nanofibrous membranes were wrapped around the Achilles tendon. (**B**) Macroscopic observations of tendons in each group. (**C**) H&E staining and Masson staining of the interface between the tendon and surrounding tissues. (**D**) Macroscopic assessments of adhesions. (**E**) Macroscopic shape assessments. (**F**) Histological assessments of adhesions. (**G**) Histological assessments of the inflammatory response. The data are presented as the mean ± standard deviation from four experimental replicates. Statistical analysis was performed by one-way ANOVA with Tukey’s post hoc analysis. *p < 0.05; **p < 0.01, ***p < 0.001; ns indicates no significance

According to the in vitro and subcutaneous experiments, we demonstrated that PEG-modified aligned nanofibers exhibited better host compatibility and were able to reduce macrophage-mediated immune responses by decreasing the expression of inflammatory genes and activation of the NLRP3 inflammasome. Therefore, we considered that the membranes could be used to prevent adhesion. For this purpose, we employed a rat model of adhesion after Achilles tendon injury (Fig. 7A) and divided the animals into four groups: the control, PCL, L-PECL, and H-PECL groups. The animals were sacrificed three weeks after surgery, and peritendinous adhesions were evaluated by gross observation and tissue staining.

As shown in Fig. 7B, severe peritendinous adhesions developed after Achilles tendon injury. The adhesions were reduced in all the groups that were treated with fibrous membranes. However, substantial amounts of adherent tissue were still observed in the PCL group. In contrast, adhesions were alleviated in both the L-PECL group and the H-PECL group, with easier separation between the tendon and surrounding tissue. We scored the peritendinous adhesions macroscopically, and the H-PECL group had the lowest score (Fig. 7D).

Subsequently, we performed H&E and Masson staining (Fig. 7C). Dense fibrous tissue was observed around the tendons in the control and PCL groups, which was consistent with general observations. The H-PECL group had the fewest adhesions, and the membranes appeared to have a sparse multilayer structure, which may be due to degradation in vivo. This contributed to the free gliding of the tendon. We performed histological scoring of peritendinous adhesions and inflammation (Fig. 7F, G), and the H-PECL group showed the best antiadhesive effect and the lowest inflammatory response.

### *Evaluation of persistent inflammatory responses in response to the nanofibrous membranes*

**Fig. 8:**
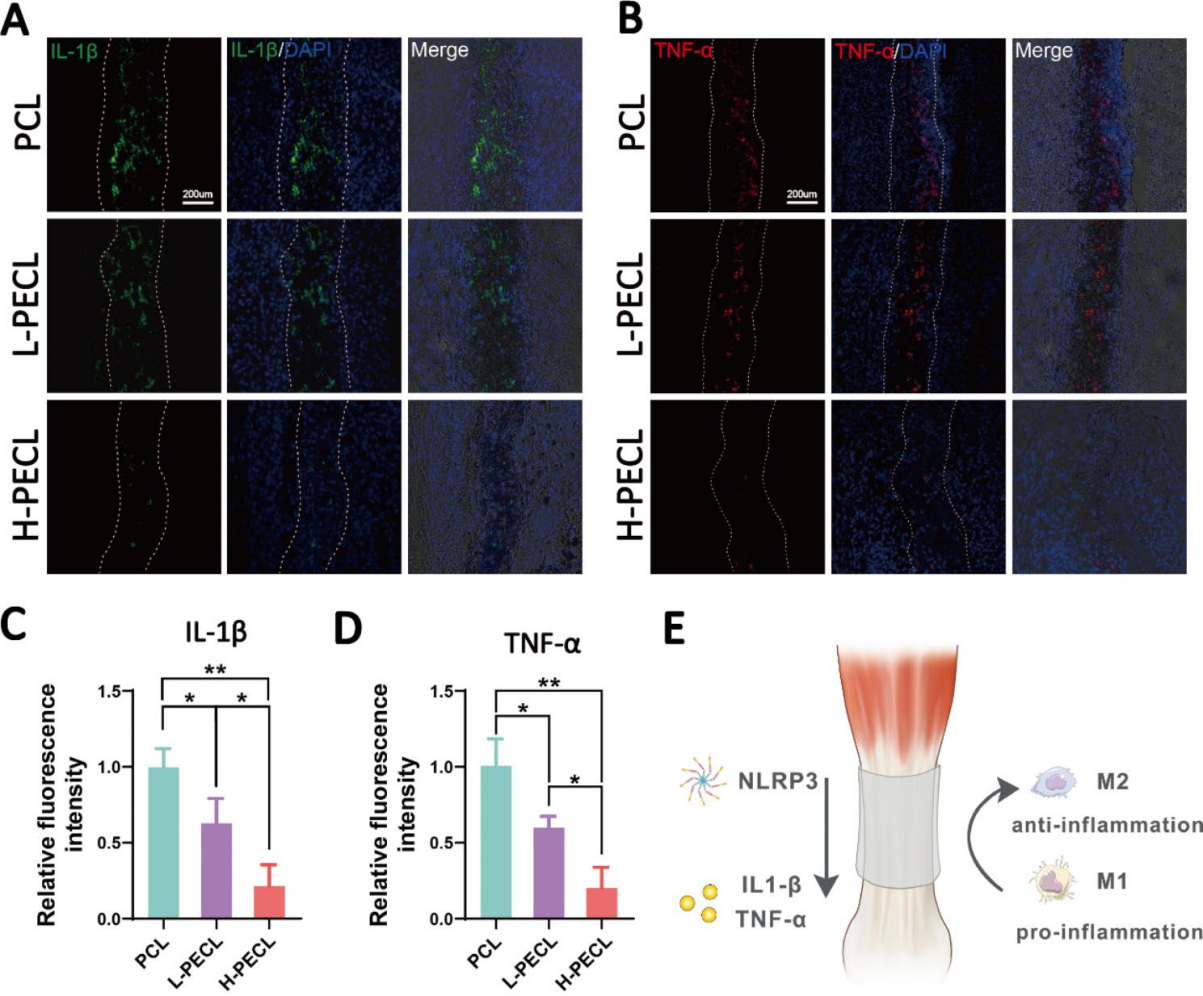
Evaluation of inflammatory responses elicited by the nanofibers at 3 weeks post-surgery. Immunofluorescence analysis of the inflammatory factors (**A**) IL-1b and (**B**) TNF-a in the implanted nanofibers. Fluorescence intensity analysis of (**C**) IL-1b and (D) TNF-α expression. (**D**) Conceptual graphs of the immune microenvironment of tendons. The data are presented as the mean ± standard deviation from four experimental replicates. Statistical analysis was performed by one-way ANOVA with Tukey’s post hoc analysis. *p < 0.05; **p < 0.01, ***p < 0.001; ns indicates no significance

We performed immunofluorescence staining for inflammatory factors to evaluate persistent inflammatory responses after implanting different types of nanofibrous membranes. The PCL group exhibited positive IL-1β and TNF-α staining, the L-PECL group exhibited weaker staining, and the H-PECL group had the weakest inflammatory factor expression (Fig. 8). This indicated that H-PECL nanofibers elicited a minimal inflammatory response after implantation and were therefore superior to PCL fibers in terms of immune compatibility and modulation (Fig. 8D).

## Discussion

FBRs to biomaterial implants are difficult problems to overcome. Implanting PCL materials often causes an inflammatory response that may develop into chronic sterile inflammation [4, 6, 28]. A recent study showed that aligned collagen fibers induced the elongation and polarization of macrophages toward the M2 phenotype [5]. These authors suggested that the structure, but not the biochemical properties, of the collagen fibers played a dominant role in this process. Another study showed that disordered nanofibers triggered M1 macrophage polarization to a greater extent than aligned nanofibers [17]. Such topographical alterations significantly affect macrophage morphology. Additionally, the surface wettability of a material also exerts a regulatory effect on macrophages. Hydrophobic surfaces are generally thought to induce M1 polarization, while hydrophilic surfaces inhibit macrophage adhesion and induce M2 macrophage polarization [29]. Our study demonstrated that simultaneous modification of the hydrophilicity and topography of implants helped mitigate foreign body reactions and further actively modulate the immune response. With the increase of hydrophilicity of aligned nanofibers, the inflammatory gene expression of macrophages adhering to them was downregulated, and M2 polarization was induced. We analyzed the cytoskeleton of macrophages seeded on the membrane and found a decrease in stress fibers as the amount of PEG increased. Similarly, it has been demonstrated that the cytoskeleton plays an important role in topography-induced macrophage polarization [30].

However, designing immunomodulatory materials for use in different applications remains difficult, and the underlying regulatory mechanisms are not clear. Here, our study found for the first time that hydrophilic nanofibers with an aligned topography can modulate the polarization of macrophages by inhibiting NLRP3 inflammasome activation, thereby regulating the immune response of the microenvironment. Inflammasomes play a critical role in the innate immune response. When danger signals are received, inflammasomes assemble and subsequently cleave inflammatory cytokine precursors to promote their maturation and release [31]. The implantation of biomaterials may disrupt tissue homeostasis and cause inflammatory responses. Studies have suggested that inflammasomes are involved in such foreign body response processes [32]. We also demonstrated in our experiments that the NLRP3 inflammasome assembled in macrophages after the subcutaneous implantation of fibrous membranes. Moreover, NLRP3 was recently reported to be associated with tissue regeneration and repair [33]. Appropriate activation and resolution of inflammasomes is necessary [20, 34]. Excessive inflammasome activation leads to prolonged inflammation and eventually fibrosis [20]. We reported here that PECL nanofibers were able to regulate NLRP3 inflammasome activation. This may be due to their aligned and hydrophilic properties. As a result, the fabricated fibrous membranes elicited a milder macrophage-mediated foreign body response. Furthermore, greater macrophage polarization toward the M2 phenotype may favor local tissue homeostasis and benefit the immune microenvironment.

The importance of macrophages in the field of biomaterial implantation cannot be overstated. Avoidance of excessive macrophage-mediated foreign body reactions is a prerequisite for the successful integration of biomaterials [4, 6]. However, in the field of tissue repair, the role of macrophages is sophisticated. Macrophages accumulate in the early stages of tissue damage, exhibiting an inflammatory phenotype, and they can then recruit many types of cells and initiate the repair process [11]. However, this process should be altered in due course, with the conversion of macrophages into M2-type cells that exhibit anti-inflammatory and pro-regenerative properties [12]. Such an appropriate shift is a difficult aspect of immunomodulatory biomaterial design, where our study provides ideas for this purpose.

## Conclusion

In this study, we demonstrated that hydrophilicity significantly contributed to material-cell interactions on the basis of topology cues from the nanofibers. This result suggested that the crosstalk between biomaterials and the immune system was multifactorial. To prove this, we prepared hydrophilic nanofibers with an aligned topography by incorporating PEG and PCL using axial electrospinning. The hydrophilic nanofibers with an aligned topography exhibited better immunomodulatory properties and compatibility. The PECL fibers allowed the recruitment of macrophages and induced macrophage polarization toward the M2 phenotype. We further presented clear evidence that the NLRP3 inflammasome is the cellular sensor by which macrophages recognize biomaterials. The NLRP3 inflammasome appeared to act as a regulator between the macrophage-mediated foreign body response and implant integration and shaped the peri-implantation immune microenvironment. These findings may guide the future design of immunomodulatory materials.

### Electronic supplementary material

Below is the link to the electronic supplementary material.

Supplementary Material 1
