# Supplementary material for: Hydrophilic nanofibers with aligned topography modulate macrophage-mediated host responses via the NLRP3 inflammasome
Source: J Nanobiotechnology. 2023 Aug 14;21:269. doi: 10.1186/s12951-023-02024-9 (PMC10424429; doi:10.1186/s12951-023-02024-9)
Supplement: Supplementary file 1 — Supplementary Material 1 [file 12951_2023_2024_MOESM1_ESM.pdf]

# Supplementary Materials

## Hydrophilic nanofibers with aligned topography modulate macrophage-mediated host responses via the NLRP3 inflammasome

*Yiming Ren* [a, b, 1], *Yi Chen* [a, b, c, 1], *Wei Chen* [a, b, 1], *Haotian Deng* [a, b], *Peiqi Li* [a, b], *Yubo Liu* [a], *Cangjian Gao* [b], *Guangzhao Tian* [a, b], *Chao Ning* [b], *Zhiguo Yuan* [b], *Xiang Sui* [b], *Shuyun Liu* [a, b, *], *Quanyi Guo* [a, b, *]

[a] *School of Medicine, Nankai University, Tianjin 300071, China.*

[b] *Institute of Orthopedics, First Medical Center, Chinese PLA General Hospital; Beijing Key Laboratory of Regenerative Medicine in Orthopedics; Key Laboratory of Musculoskeletal Trauma and War Injuries PLA, No. 28 Fuxing Road, Haidian District, Beijing 100853, China.*

[c] *Department of Plastic and Reconstructive Surgery, The First Medical Center, Chinese PLA General Hospital, Beijing, 100853, China.*

[1] These authors contributed equally to the work.

* Corresponding author. E-mail address: clear_ann@163.com (S. Liu), doctorguo_301@163.com (Q. Guo).

**Supplementary Materials and Methods:**

1.1. RNA isolation and quantitative real-time PCR

For ensuring that only macrophages cultured on the scaffold were included in the experiments, we transferred the nanofibrous membrane containing BMDM into another plate. The cells were digested from the membrane. Total mRNA was extracted by adding Trizol reagent (Invitrogen) according to the manufacturer's instructions. To analyze gene expression levels in macrophages, cDNA was reverse transcribed from mRNA using a PrimeScript RT kit (TOYOBO, Japan) and then analyzed by real-time fluorescent quantitative PCR (qPCR) (StepOnePlus, ThermoFisher Scientific). The primer sequences are shown in the Supplement Table S1.

1.2. Western Blot

Cells were lysed by RIPA buffer containing protease and phosphatase inhibitors (Solarbio, China). Proteins from lysates were then separated by sodium dodecyl sulfate polyacrylamide gel electrophoresis (SDS-PAGE), and then electrotransferred to polyvinylidene fluoride (PVDF) membranes. PVDF membranes were blocked with 5% skim milk for 1 h at room temperature and then incubated with primary antibodies overnight at 4°. On the following day, the membranes were incubated with HRP-coupled secondary antibodies for 1 h at room temperature. Finally，imagination was performed with a chemiluminescence imaging system(G: BOX Chemi-XRQ, Syngene, UK). Primary antibodies included anti-IL 1β (Abcam), anti-Caspase 1 (Novus), anti-NLRP3 (Novus), anti-NF κB (CST). β-actin (Santa Cruz) was used as an internal control.

1.3. Macroscopic evaluation

A 5-level classification system was devised to estimate the seriousness of tendon adhesions and macroscopic shapes as Supplement Table S2, S3.

1.4. In vivo immunofluorescence staining

For immunofluorescence staining of Achilles tendon tissue, we hydrated the tissue sections and fixed them with 4% paraformaldehyde for 10 min. After washing, the tissue was incubated in PBS containing 5% goat serum and 2% BSA for 1 h at room temperature. Then primary antibody was added for incubation overnight at 4°C. Upon washing with PBS, fluorescent-coupled secondary antibody was added for 1 h at room temperature. DAPI was then used to stain cell nuclei. A fluorescence microscope (Nikon, Japan) was employed for fluorescence observation and photography. Primary antibody: IL-1β (Abcam), Arg-1 (Abcam), ASC (Novus).

**Supplementary Tables:**

Table S1. Sequences of primers for qRT-PCR assay.

| | | |
|---|---|---|
| IL-1b | forward primer | TACAGGCTCCGAGATGAACA |
| | reverse primer | AGGCCACAGGTATTTTGTCG |
| IL-6 | forward primer | CTGCAAGAGACTTCCATCCAG |
| | reverse primer | AGTGGTATAGACAGGTCTGTTGG |
| TNF-a | forward primer | GGTCTGGGCCATAGAACTGA |
| | reverse primer | CAGCCTCTTCTCATTCCTGC |
| IL-10 | forward primer | GCTCTTACTGACTGGCATGAG |
| | reverse primer | CGCAGCTCTAGGAGCATGT |
| GAPDH | forward primer | CTTTGTCAAGCTCATTTCCTGG |
| | reverse primer | TCTTGCTCAGTGTCCTTGC |

Table S2.

| GRADE | Macroscopic assessments of adhesions |
|---|---|
| 1 | No adhesions |
| 2 | Adhesions can be released by blunt dissection |
| 3 | ≤50% of the area that must be released by sharp dissection |
| 4 | 51-97.5% of the area that must be released by sharp dissection |
| 5 | ≥97.5% of the area that must be released by sharp dissection |

Table S3.

| GRADE | Macroscopic shapes assessments |
|---|---|
| 1 | Intensely thickened, with palpable swelling and redness |
| 2 | Intensley thickened, with palpable swelling and no redness |
| 3 | Moderately thickened, with palpable swelling and no redness |
| 4 | Moderately thickened, without swelling or redness |
| 5 | Normal/Slightly thickened, without swelling or redness |

Table S4.

| GRADE | Histologic assessments of adhesions |
|---|---|
| 1 | No adhesions |
| 2 | Mild (adhesions on the surface of the tendon <33%) |
| 3 | Moderate (adhesions on the surface of the tendon were 33-66%) |
| 4 | Severe (adhesions on the surface of the tendon were >66%) |

Table S5.

| GRADE | Inflammatory responses assessments |
|---|---|
| 1 | Almost no lymphocytes |
| 2 | Partially infiltrated with lymphocytes |
| 3 | Partially infiltrated with macrophages and a small amount of neutrophils |
| 4 | Marked macrophage and inflammatory response with a noticeable infiltration of neutrophils |

Table S6.

| Antibodies | Source | Dilution |
|---|---|---|
| iNOS | Abcam | 1:500 |
| Arg-1 | Abcam | 1:200 |
| ASC | Novus | 1:60 |
| CD68 | Novus | 1:100 |
| CD86 | Novus | 1:200 |
| CD206 | Cell Signaling | 1:500 |

**Supplementary Figure:**

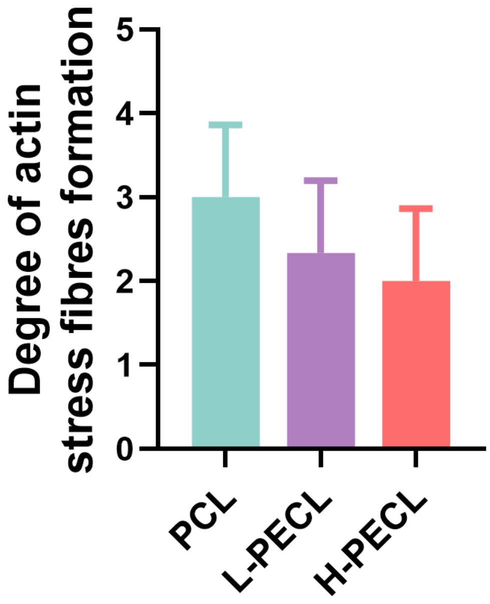


Figure S1. Degree of actin stress fiber formation in macrophages cultured on the different nanofiber membranes.
